# EVALUATION OF A DECADE OF ONCOLOGICAL-ORTHOPEDIC PROCEDURES IN BRAZIL (2015–2024) AND THE IMPACT OF COVID-19

**DOI:** 10.1590/1413-785220253303e297250

**Published:** 2025-12-01

**Authors:** Alex Guedes, Olavo Pires de Camargo, Ediriomar Peixoto Matos, Mario Castro Carreiro, Felype Figueiredo Rios, Antônio Henrique Santos Guimarães, Kleber Antas Meyer, Nayara Fulgêncio Leite de Lima, Bruno Garcia Barreto, Enilton de Santana Ribeiro de Mattos, César Romero Antunes, Eduardo Silva Reis Barreto

**Affiliations:** 1 Universidade Federal da Bahia, Faculdade de Medicina da Bahia, Departamento de Cirurgia Experimental e Especialidades Cirurgicas, Salvador, BA, Brazil.; 2 Santa Casa de Misericordia da Bahia, Hospital Santa Izabel, Grupo de Oncologia Ortopedica, Salvador, BA, Brazil.; 3 Universidade Federal da Bahia, Orthopedic and Traumatology Research Group, Salvador, BA, Brazil.; 4 Universidade Federal da Bahia, Programa de Residencia Medica em Ortopedia e Traumatologia, Salvador, BA, Brazil.; 5 Universidade de Sao Paulo, Faculdade de Medicina, Hospital das Clinicas, Instituto de Ortopedia e Traumatologia, Sao Paulo, SP, Brazil.

**Keywords:** Bone Neoplasms, Prostheses and Implants, Orthopedic Procedures, Unified Health System, COVID-19, Neoplasias Ósseas, Próteses e Implantes, Procedimentos Ortopédicos, Sistema Único de Saúde, COVID-19

## Abstract

**Objective:**

To evaluate the regional distribution of hospital admission authorizations (AIH), total and average hospitalization cost (AHC), average length of stay, number of deaths and mortality rate related to oncological-orthopedic procedures funded by the Unified Health System (SUS) between 2015 and 2024, with an emphasis on the impact of the COVID-19 pandemic.

**Methods:**

Ecological study with time series based on data obtained from the SUS Hospital Information System, analyzed by Brazilian regions in the pre-pandemic, pandemic and post-pandemic periods. Regional differences were calculated using ANOVA or Kruskal-Wallis. The impact of the pandemic was analyzed using T-tests and ARIMA with intervention. Statistical analysis was performed in R.

**Results:**

9,120 AIHs were recorded, mostly in the Southeast (4,375) and South (2,252) regions. Multiple regional variations were found for all the variables evaluated. Only the AHC was impacted – there was an increase in costs per procedure; The other variables maintained the trend after the beginning of the pandemic.

**Conclusions:**

Despite the increase in the AHC, we did not observe significant variation in the number of AIH, ALS, and MR when analyzing the pre-pandemic and pandemic periods, suggesting that there was no direct impact on the performance of the analyzed procedure. *Level of Evidence III; Retrospective*_f_ *comparative study*_e_.

## RESUMO

**Objetivo:**

Avaliar a distribuição regional das autorizações de internação hospitalar (AIH), custos total e médio de internação hospitalar (CMH), tempo médio de permanência, número de óbitos e taxa de mortalidade relacionados aos procedimentos oncológico-ortopédicos financiados pelo Sistema Único de Saúde (SUS) entre 2015 e 2024, com ênfase no impacto da pandemia de COVID-19.

**Métodos:**

Estudo ecológico com séries temporais baseadas em dados obtidos do Sistema de Informações Hospitalares do SUS, analisados por regiões brasileiras, nos períodos pré-pandemia, pandêmico e pós-pandemia. As diferenças regionais foram calculadas por ANOVA ou Kruskal-Wallis. O impacto da pandemia foi analisado por meio de testes T e ARIMA com intervenção. A análise estatística foi realizada no R.

**Resultados:**

Foram registradas 9.120 AIHs, a maioria nas regiões Sudeste (4.375) e Sul (2.252). Múltiplas variações regionais foram encontradas para todas as variáveis avaliadas. Apenas o CMH foi impactado – houve aumento nos custos por procedimento; as demais variáveis apresentaram manutenção da tendência após o início da pandemia.

**Conclusões:**

Apesar do aumento do CMH, não foi observada variação significativa no número de AIH, TMP e TM quando analisados os períodos pré-pandêmico e pandêmico, sugerindo que não houve impacto direto no desempenho do procedimento analisado. *Nível de Evidência III; Estudo*_e_ *Comparativo Retrospectivo*_f_.

## INTRODUCTION

The treatment of bone tumors through resection followed by prosthetic replacement or reconstruction with fixation has revolutionized orthopedics. Advances in medical and implant technologies have contributed to improved outcomes, both oncological and functional, leading to enhanced quality of life for patients.^1^

The Hospital Information System (SIH) of Brazil's Unified Health System (SUS) provides a comprehensive database for analyzing hospital admissions across the country.^2^ Studying the regional distribution of procedures funded by SUS is essential for guiding effective healthcare strategies, enabling the identification of potential challenges and opportunities for improving care for patients with bone neoplasms.

The COVID-19 pandemic imposed major challenges on healthcare systems worldwide, affecting the capacity to perform elective procedures, including bone tumor resection surgeries,^3^ due to the reallocation of resources toward managing COVID-19.^4^ To date, the impact of the COVID-19 pandemic on resection procedures involving prosthetic replacement (endoprosthesis) or reconstruction with fixation within the SUS system has not been evaluated.

This study aimed to describe the regional distribution of hospital admission authorizations (AIH), total hospitalization costs (THC), average hospitalization costs (AHC), average length of stay (ALS), number of deaths (ND), and mortality rate (MR) related to resection procedures with prosthetic replacement (endoprosthesis) or reconstruction with fixation in oncology, funded by SUS over a ten-year period (2015-2024), with particular emphasis on the impact of the COVID-19 pandemic.

## METHODS

This ecological time-series study analyzed bone tumor resection procedures involving either prosthetic replacement (endoprosthesis) or reconstruction with fixation in oncology, categorized under code 04.16.09.010-9 in the SUS Procedure, Medication, Orthoses, Prostheses, and Special Materials Management System (SIGTAP). The primary focus was to assess the potential impact of the COVID-19 pandemic. Epidemiological data were examined for the period from January 1, 2015, to December 31, 2024, encompassing three distinct phases: pre-pandemic (January 2015 to January 2020), pandemic (February 2020 to May 2022), and post-pandemic (June 2022 to December 2024).

Data were obtained from the SUS Hospital Information System (SIH/SUS),^2^ which compiles information on procedures performed within Brazil's public healthcare system. Analyses were stratified by geographic region (Midwest, North, Northeast, Southeast, and South). The variables examined included the AIH, THC, AHC, ALS, ND, and MR.

### Statistical Analysis

Continuous variables were first assessed for normality using the Shapiro-Wilk test. Variables with normal distributions were summarized as means and standard deviations, while non-normally distributed data were presented as medians and interquartile ranges (Q1–Q3).

Comparisons across the three time periods were performed using Student's T-test for normally distributed variables, accounting for variance differences where applicable. For non-normal data, the Mann-Whitney U test was applied. Regional comparisons were conducted using one-way ANOVA for normally distributed variables or the Kruskal-Wallis test for non-normal data.

To analyze temporal trends and detect structural changes potentially associated with the onset of the pandemic, autoregressive integrated moving average models with intervention (intervention ARIMA) were employed. The intervention point was set in February 2020. The auto.arima function was used to automatically identify optimal model parameters (p, d, q) based on the corrected Akaike Information Criterion (AICc). These models estimated the pandemic's impact on time series levels while adjusting for trend, seasonality, and autocorrelation components.

Model adequacy was assessed through residual analysis, including the Ljung-Box test for serial autocorrelation and first-order autocorrelation inspection (ACF1). Predictive accuracy was quantified using the Mean Absolute Percentage Error (MAPE), calculated by comparing observed values with model-adjusted estimates.

All analyses were conducted using R software (version 4.3.1), employing the forecast, tibble, lubridate, dplyr, and ggplot2 packages. A p-value of <0.05 was considered statistically significant for all tests.

### Ethical Considerations

Ethics committee approval was not required for this study, as it used secondary data derived from publicly available sources, in accordance with Resolution No. 510/2016 of the Brazilian National Health Council.

## RESULTS

During the study period, a total of 9,120 hospital admission authorizations (AIHs) were issued under SIGTAP-SUS code 04.16.09.010-9, distributed as follows: 545 in the Midwest, 1,463 in the Northeast, 485 in the North, 4,375 in the Southeast, and 2,252 in the South. Regional analyses of AHC, ALS, and MR are presented in Table 1.

**Table 1:** Regional comparison of hospital outcomes in the SUS: number of procedures, cost, length of stay and mortality rate (2015–2024).

	North	Northeast	Southeast	South	Midwest	Total	p
Number of procedures (median Q1 – Q3)	4.0 (2.0 - 6.0)	12.0 (8.0 - 16.0)	37.0 (32.3 - 42.0)	19.0 (15.0 - 23.0)	4.0 (2.0 - 6.0)	78.0 (68.00 - 87.75)	<0.001
Mean value (R$) (mean ± standard deviation)	9025.37 (± 3532.30)	8363.57 (± 2220.35)	7683.34 (± 986.04)	9700.71 (± 1624.23)	6896.75 (± 2343.40)	8296.15 (± 985.86)	<0.001
Length of stay in days (median, Q1 – Q3)	10.0 (6.9 - 13.5)	4.40 (3.68 - 5.60)	7.0 (6.2 - 8.2)	7.25 (6.33 - 8.70)	4.60 (3.30 - 6.90)	6.90 (6.40 - 7.68)	<0.001
Mean mortality rate (%) (median, Q1 – Q3)	0.0 (0.0 - 0.0)	0.0 (0.0 - 5.88)	2.60 (0.0 - 5.71)	4.35 (0.0 - 7.34)	0.0 (0.0 - 0.0)	3.53 (2.18 - 5.14)	<0.001

The THC for SUS during the period was BRL 78,317,313.75, with an estimated AHC of BRL 8,296.15 (±985.86). Considerable variation was observed among regions. The Midwest had the lowest average monthly AHC (BRL 6,896.75 ± 2,343.40), while the South recorded the highest (BRL 9,700.71 ± 1,624.23). Statistically significant differences were found across regional combinations. The South showed significantly higher AHC values than all other regions, except for the North (p = 0.25). Conversely, the Midwest exhibited significantly lower AHCs, especially in comparison with the South, North, and Northeast.

The national average ALS was 7.13 days, but substantial regional variability was observed. The North reported the highest average (11.90 days; IQR: 6.90–13.50), while the Northeast had the lowest (4.98 days; IQR: 3.68–5.60). Statistical tests identified significant differences across multiple regional comparisons. The North had significantly longer ALSs than all other regions. In contrast, the Northeast had significantly shorter ALSs compared to the North, South, and Southeast, indicating a trend of shorter hospital stays in that region.

During this period, 357 deaths were recorded. The overall MR in Brazil was 3.77 deaths per 100 hospitalizations, with notable regional differences. The Northeast had the lowest MR (2.79%), whereas the South registered the highest (4.86%). Significant differences were found between some regional pairs. Specifically, the South had a significantly higher MR than the North, Northeast, and Southeast. Additionally, the Midwest presented a significantly higher MR compared to the Northeast.

**Figure 1:**
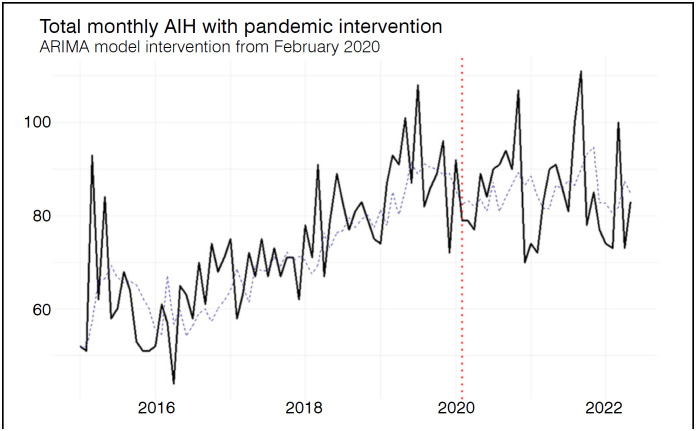
Monthly average number of authorized hospital admissions (AIH). Temporal trends with intervention point (red dotted line) referring to the beginning of the COVID-19 pandemic.

Source: Authors.

In the pre-pandemic period, the mean number of monthly authorized AIHs was 72.51 (±14.01), increasing to 85.04 (±10.63) during the pandemic, with a statistically significant difference (p < 0.001). In the time series analysis with intervention, ARIMA (0,1,1)(1,0,0)[12], the estimated intervention coefficient was −7.76 and not statistically significant (p = 0.216), indicating no sustained structural change in the series level associated with the onset of the pandemic. Residual analysis confirmed adequate model fit, with no significant autocorrelation (Ljung-Box test: Q = 12.89; df = 16; p = 0.681), near-zero first-order autocorrelation (ACF1 = −0.017), and a MAPE of 10.46%, indicating satisfactory forecasting performance. Figure 1 illustrates the variation in the number of AIHs over the study period.

**Figure 2:**
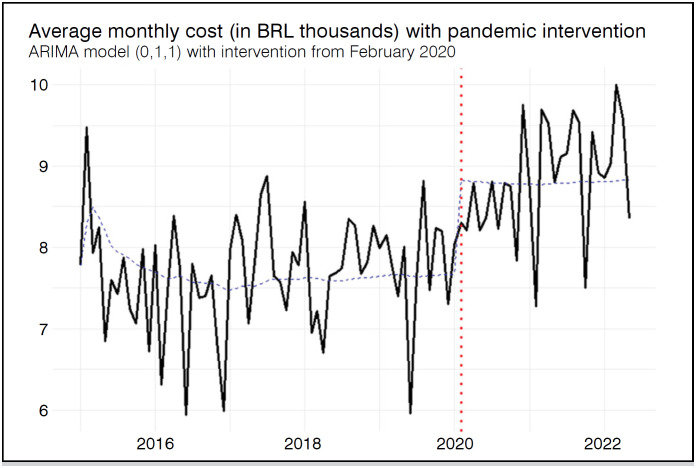
Variation in average monthly hospitalization cost over time (in thousands of BRL). Temporal trends with intervention point (red dotted line) referring to the beginning of the COVID-19 pandemic.

Source: Authors.

During the pre-pandemic period, the average monthly AHC was BRL 7,670.90 (±691.02), increasing to BRL 8,828.04 (±691.13) during the pandemic, a statistically significant difference (p < 0.001). To investigate the temporal dynamics and estimate the structural impact of the pandemic, an intervention time series model was applied. In the ARIMA (0,1,1), the intervention was associated with an estimated level increase of BRL 1,157.22, which was statistically significant (p < 0.001). Residual analysis indicated adequate model fit, with no signs of autocorrelation (Ljung-Box test: Q = 17.46; df = 17; p = 0.4235), near-zero first-order autocorrelation (ACF1 = 0.03), and a MAPE of 6.82%. Figure 2 shows the variation in AHC over the study period.

**Figure 3:**
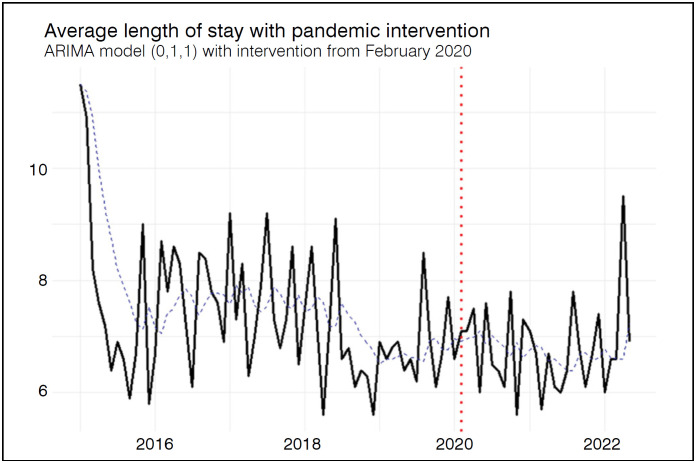
Average length of stay, in days. Temporal trends with intervention point (red dotted line) referring to the beginning of the COVID-19 pandemic.

Source: Authors.

In contrast, a direct comparison between pre-pandemic and pandemic periods showed that median ALS decreased from 7.00 days (IQR: 6.60–8.25) to 6.70 days (IQR: 6.10–7.25), a statistically significant difference (p = 0.027). However, the ARIMA (0,1,1) intervention model did not identify a significant structural change associated with the pandemic onset. Residual analysis confirmed no significant autocorrelation (p = 0.2861; ACF1 = 0.114), and the estimated intervention coefficient was 0.0330 (p = 0.961), indicating no significant effect. These results suggest that, despite the observed difference in medians, there was no sustained change in the trend of hospitalization over time. Figure 3 shows the variation in ALS and the difference between pre-pandemic and pandemic periods.

**Figure 4:**
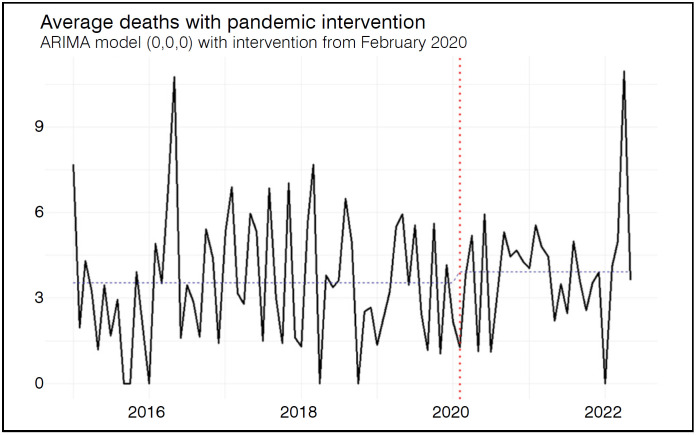
Mean monthly in-hospital mortality rate. Temporal trends with intervention point (red dotted line) referring to the beginning of the COVID-19 pandemic.

Source: Authors.

Regarding monthly MR, median values rose from 3.23 (IQR: 1.63–5.37) in the pre-pandemic period to 3.98 (IQR: 2.75–4.96) during the pandemic, but the difference was not statistically significant (p = 0.344). The ARIMA (0,0,0) model with intervention also did not detect a structural change associated with the onset of the pandemic. The estimated intervention coefficient was 0.3807 (p = 0.433). The lack of statistical significance supports the interpretation that the pandemic did not consistently impact the level of the mortality rate series. Residual analysis confirmed adequate model performance (Ljung-Box test: Q = 20.58; df = 17; p = 0.195), although the predictive accuracy was lower (MAPE = 54.88%), indicating greater variability and reduced forecasting precision for this indicator. Figure 4 illustrates the variation in MR over time and the comparison between pre-pandemic and pandemic periods.

A comparative analysis between pre- and post-pandemic periods was also conducted to assess changes in the studied indicators after the onset of the pandemic. The mean monthly number of AIHs increased from 72.51 (14.01) in the pre-pandemic period to 79.71 (17.28) in the post-pandemic period, with a significant difference (p = 0.034). The average monthly AHC also increased substantially, from BRL 7,670.90 (691.02) to BRL 9,046.05 (911.23), a highly significant difference (p < 0.001). Regarding ALS, there was a slight median reduction from 7.00 days (IQR: 6.60–8.25) to 6.80 days (IQR: 6.40–7.40), which was not statistically significant (p = 0.388). Similarly, the median monthly MR increased from 3.23 (IQR: 1.63–5.37) to 4.08 (IQR: 2.38–5.88), but this difference also did not reach statistical significance (p = 0.254).

## DISCUSSION

Until 2013, orthopedic-oncologic procedures funded by the SUS did not include reconstruction or fixation; they were limited to the replacement of affected segments with non-conventional endoprostheses—the only compatible implants listed in SIGTAP/SUS at that time. Following a proposal by one of the authors of this article, the Brazilian Ministry of Health approved a change to the procedure coding system through Ordinance No. 2,947 of December 21, 2012, which was implemented the following year. This revision expanded the range of allowable implants to include conventional and specialized plates, rods, and external fixators, enabling the performance of reconstruction and fixation procedures in addition to replacements.

We found a national ALS of 7.13 days, with significant variation across Brazilian regions. In the United States, the ALS for patients undergoing resection with endoprosthetic replacement is approximately 8 days, whereas in the United Kingdom, it reaches around 15 days.^5,6^ Regional differences in ALS may reflect factors such as case complexity, rehabilitation protocols, and availability of postoperative care.

In our time series, the average monthly AHC was estimated at BRL 8,296.15, with wide regional variation. This value is substantially lower than in the United States, where the cost of a single non-conventional endoprosthesis can exceed USD 50,000.^7^ Several factors contribute to lower costs within SUS: (i) public funding through tax-based financing, which reduces out-of-pocket expenses for patients; (ii) government price regulation via SIGTAP-SUS, which sets reimbursement limits for procedures and hospitalizations; (iii) operational efficiency in public hospitals, aimed at maximizing patient volume and minimizing waste; (iv) lower labor costs compared to high-income countries; and (v) economies of scale, as SUS serves millions of users, enabling bulk purchasing at reduced prices. Costs related to multiple surgeries performed in a single session and postoperative care in intensive units were not included in this study's cost calculations.

ND analysis revealed uneven distribution across states, with higher MR in the South and lower MR in the Northeast. The national MR associated with these procedures in Brazil (4.86%) is comparable to rates reported in other countries, varying by case complexity, patient age, and quality of postoperative care.^8^ U.S. studies report MR rates as high as 9% for bone resection surgeries with endoprosthetic reconstruction.^9^

During the COVID-19 pandemic, global restrictions on healthcare resources significantly affected elective surgeries, reshaping orthopedic care. Initially, elective orthopedic procedures were suspended, later resuming under stricter safety protocols covering hospitalization through postoperative rehabilitation. In Italy, these surgeries were suspended starting February 23, 2020;^10^ the American College of Surgeons recommended halting all elective surgeries in March 2020 to reduce exposure and conserve hospital resources.^11,12^ The United Kingdom issued similar guidance on April 15, 2020, aiming to ease bed demand and support the pandemic response.^13^ As the health crisis evolved, new safety protocols enabled a gradual resumption of elective surgeries and outpatient services.

During the first two years of the COVID-19 pandemic in Brazil (2020–2021), a sharp decline was observed in the number of AIHs authorized for primary total knee arthroplasty, with reductions exceeding 50% in some regions.^14^ A similar trend was noted in total hip arthroplasty (THA); in 2020, the impact of the pandemic led to a 28.6% drop in primary THA procedures, with an even greater reduction (46.3%) in elective cases. In contrast, THAs carried out on an urgent basis—mostly related to fractures—remained stable during this period.^15^ For shoulder arthroplasties funded by SUS, a 6% decrease in authorized AIHs was observed between 2020 and 2021, with the Midwest and Northeast regions being the most affected, showing reductions of 45.5% and 16.5%, respectively.^16^

In most countries, only essential activities and COVID-19 treatment were prioritized.^17^ Bone tumors represent high-morbidity conditions that require timely and optimal multidisciplinary treatment, as delays or interruptions may pose life-threatening risks. In Brazil, the National Health Surveillance Agency (ANVISA), through Technical Note 06/2020, recommended prioritizing essential elective surgeries during the COVID-19 pandemic, particularly oncologic procedures due to their high risk of complications.^18^ Time series analysis showed that, during the pandemic, the previously observed upward trend in the monthly average number of AIHs continued. This may explain why, unlike other high-complexity orthopedic procedures funded by SUS, the trend of increasing AIHs for the procedures analyzed was maintained throughout the pandemic period.

Thaler et al. (2020)^19^ investigated the potential impact of the COVID-19 pandemic on the diagnostic workup and treatment of patients with musculoskeletal tumors through an online survey of members of the International Society of Limb Salvage and the European Musculo-Skeletal Oncology Society. They reported a substantial global reduction in procedures, with up to 20% of respondents indicating suspension or postponement of treatments, leading to longer waiting times and potentially worse clinical outcomes. Similarly, Onesti et al. (2022)^20^ examined the impact of the pandemic on the diagnosis and treatment of patients with soft tissue and bone sarcomas or aggressive benign musculoskeletal diseases at a single referral center in Italy. They observed an average diagnostic delay of approximately 13 days during the first year of the pandemic compared to the pre-pandemic period.

Although significant differences were observed between the pre-pandemic and pandemic periods for both AHC and ALS, time series models with intervention indicated that only the AHC was significantly affected by the pandemic, showing a marked increase. This suggests a real rise in hospitalization complexity and costs, possibly due to more severe cases or changes in hospital management during the pandemic. In contrast, the reduction in ALS appears to be a temporary fluctuation without a sustained trend. No significant differences or trends were identified for mortality. These findings indicate that the pandemic's impact was heterogeneous, more pronounced in indicators related to care complexity than in those linked to length of stay or short-term clinical outcomes.

The limitations of this study are consistent with those of other retrospective reviews using databases. Most are related to underreporting of cases, lack of information on the sociodemographic characteristics of the affected population, heterogeneity across Brazilian regions and states, and unavailability of specific data on underlying neoplasms, comorbidities, and causes of death. The data collected refers exclusively to the hospitalization period, making it impossible, for instance, to assess postoperative mortality. Additionally, the mortality model did not demonstrate an adequate fit, which limits the interpretation of the time trend for this variable, despite the clear absence of differences across the pre-pandemic, pandemic, and post-pandemic periods.

## CONCLUSION

We found no significant reduction in hospital admissions for bone tumor resection with prosthetic replacement or reconstruction and fixation during the COVID-19 pandemic. Time series analysis showed a continued upward trend in the average monthly hospital authorizations and AHC. These findings suggest that the pandemic may not have significantly affected the performance of such surgical procedures, possibly indicating that ANVISA's guidance to prioritize essential elective surgeries, especially oncologic ones, was followed by high-complexity oncology centers funded by the Brazilian public health system.

## References

[1] Guzik G (2016;). Results of the treatment of bone metastases with modular prosthetic replacement--analysis of 67 patients. J Orthop Surg Res.

[2] Brasil Datasus. Ministério da Saúde. [Internet]..

[3] Nishizawa M, Nagata K, Adejuyigbe B, Shinozaki T, Yamada K (2024). Trends in inpatient orthopedic surgery during the COVID-19 pandemic in Japan: a nationwide data study. BMC Musculoskelet Disord.

[4] Crawford AM, Lightsey IV HM, Xiong GX, Ye J, Call CM, Pomer A (2023). Changes in Elective and Urgent Surgery Among TRICARE Beneficiaries During the COVID-19 Pandemic. Mil Med.

[5] Kapoor S, Singh S, Bassett P, Gerrand C (2022). Predicting length of stay after proximal femoral endoprosthetic replacement for oncological conditions. Surgeon.

[6] Hughes N, Birlingmair J, Baker J, Tideman G, Sweeney K (2022). Evaluating factors affecting length of hospital stay in patients with metastatic bone tumors. J Orthop.

[7] Wilson RJ, Sulieman LM, VanHouten JP, Halpern JL, Schwartz HS, Devin CJ (2017). Cost-utility of osteoarticular allograft versus endoprosthetic reconstruction for primary bone sarcoma of the knee: A markov analysis. J Surg Oncol.

[8] Johnson JD, Satcher RL, Feng L, Lewis VO, Moon BS, Bird JE (2023). What Is the Prosthetic Survival After Resection and Intercalary Endoprosthetic Reconstruction for Diaphyseal Bone Metastases of the Humerus and Femur?. Clin Orthop Relat Res.

[9] Levin AS (2023). CORR Insights®: What is the Prosthetic Survival After Resection and Intercalary Endoprosthetic Reconstruction for Diaphyseal Bone Metastases of the Humerus and Femur?. Clin Orthop Relat Res.

[10] D’Angelo F, Monestier L, De Falco G, Mazzacane M, Stissi P (2020). Management of Traumatology Patients During the Coronavirus (COVID-19) Pandemic: Experience in a Hub Trauma Hospital in Northern Italy. Indian J Orthop.

[11] Jain A, Jain P, Aggarwal S (2020). SARS-CoV-2 Impact on Elective Orthopaedic Surgery: Implications for Post-Pandemic Recovery. J Bone Joint Surg Am.

[12] Couto RA, Wiener TC, Adams WP (2021). Evaluating Postoperative Outcomes of Patients Undergoing Elective Procedures in an Ambulatory Surgery Center During the COVID-19 Pandemic. Aesthet Surg J.

[13] Baxter I, Hancock G, Clark M, Hampton M, Fishlock A, Widnall J (2020). Paediatric orthopaedics in lockdown: A study on the effect of the SARS-Cov-2 pandemic on acute paediatric orthopaedics and trauma. Bone Jt Open.

[14] Naito GM, Pimentel CSS, Silva RR, Guedes AAL, Guedes A (2022). Primary total knee arthroplasties under the Brazilian Public Health Unic System (SUS) - Number of procedures, regional distribution, hospitalization costs, average length of hospital stay and mortality (2012-2021). Res Soc Dev.

[15] Torres TMN, Martins BK, da Silva AA, de Assunção CAA, de Mattos ESR, Guedes A (2023). PRIMARY TOTAL HIP ARTHROPLASTIES UNDER BRAZILIAN PUBLIC HEALTH SYSTEM (2012-2021). Acta Ortop Bras.

[16] Leite LMB, Figueredo LM, Barreto ESR, Leandro MP, Ejnisman B (2023). Shoulder Arthroplasties in the Brazilian Unified Health System (SUS) - Number of procedures, regional distribution, hospitalization expenses, average length of stay, and mortality (2012-2021). Res Soc Dev.

[17] De Simone B, Chouillard E, Di Saverio S, Pagani L, Sartelli M, Biffl WL (2020). Emergency surgery during the COVID-19 pandemic: what you need to know for practice. Ann R Coll Surg Engl.

[18] Brasília: Governo Federal (2020). Agência Nacional de Vigilância Sanitária (Brazil). Nota técnica GVIMS/GGTES/ANVISA No 06/2020. [Internet].

[19] Thaler M, Khosravi I, Leithner A, Papagelopoulos PJ, Ruggieri P (2020). Impact of the COVID-19 pandemic on patients suffering from musculoskeletal tumours. Int Orthop.

[20] Onesti CE, Vari S, Nardozza F, Maggi G, Minghelli D, Rossi B (2022;). The impact of the COVID-19 pandemic on diagnosis and treatment of patients with soft tissue and bone sarcomas or aggressive benign musculoskeletal diseases: A single-center retrospective study (SarCorD study). Front Oncol.

